# Molecular Characterization of a Non–*Babesia divergens* Organism Causing Zoonotic Babesiosis in Europe

**DOI:** 10.3201/eid0908.020748

**Published:** 2003-08

**Authors:** Barbara L. Herwaldt, Simone Cacciò, Filippo Gherlinzoni, Horst Aspöck, Susan B. Slemenda, PierPaolo Piccaluga, Giovanni Martinelli, Renate Edelhofer, Ursula Hollenstein, Giovanni Poletti, Silvio Pampiglione, Karin Löschenberger, Sante Tura, Norman J. Pieniazek

**Affiliations:** * Centers for Disease Control and Prevention, Atlanta, Georgia, USA; † Istituto Superiore di Sanità, Rome, Italy; ‡ University of Bologna, Bologna, Italy; § Clinical Institute of Hygiene of the University of Vienna, Vienna, Austria; ¶ University of Veterinary Medicine of Vienna, Vienna, Austria; # University Hospital for Internal Medicine I, Vienna, Austria

**Keywords:** babesiosis, *Babesia divergens*, *Babesia odocoilei*, *Babesia venatorum*, EU1, Italy, Austria, taxonomy, *Ixodes ricinus*, 18S rRNA gene, research

## Abstract

In Europe, most reported human cases of babesiosis have been attributed, without strong molecular evidence, to infection with the bovine parasite *Babesia divergens*. We investigated the first known human cases of babesiosis in Italy and Austria, which occurred in two asplenic men. The complete 18S ribosomal RNA (18S rRNA) gene was amplified from specimens of their whole blood by polymerase chain reaction (PCR). With phylogenetic analysis, we compared the DNA sequences of the PCR products with those for other *Babesia* spp. The DNA sequences were identical for the organism from the two patients. In phylogenetic analysis, the organism clusters with *B. odocoilei,* a parasite of white-tailed deer; these two organisms form a sister group with *B. divergens*. This evidence indicates the patients were not infected with *B. divergens* but with an organism with previously unreported molecular characteristics for the 18S rRNA gene.

Babesiosis is a tick-borne zoonosis caused by intraerythrocytic protozoa of the genus *Babesia* (*1*,*2*). The world’s first well-documented human case of babesiosis was a fatal case in 1956 in an asplenic man in the former Yugoslavia (*3*). Since then, hundreds of human cases of babesiosis have been reported in the United States, approximately 30 cases have been reported in Europe (*4*–*7*), and a few cases have been reported elsewhere.

Most of the reported U.S. cases have been caused by infection with *Babesia microti*, a parasite of small mammals transmitted by *Ixodes scapularis* ticks. Most European cases have been attributed to *B. divergens*, a parasite of cattle transmitted by *I. ricinus*. However, from 1991 through 2000, additional zoonotic *Babesia* and *Babesia*-like pathogens have been identified and characterized with molecular techniques. These pathogens include, in the United States, the WA1- (for “Washington 1”) and CA1- (for “California 1”) type parasites and the MO1 (for “Missouri 1”) parasite (*8*–*10*) and, in Europe, the organism we describe here.

We report what to our knowledge are the first described human cases of babesiosis in Italy and Austria and provide evidence that the etiologic agent of the two cases, which is related to but clearly not *B. divergens,* has molecular characteristics that have not previously been reported. Following the precedent we previously established for reports of newly characterized organisms in the United States, we refer here to this organism from Europe as EU1 (for “European Union 1”).

## Methods

### Serologic Testing

Serum specimens from the patients were tested at the Centers for Disease Control and Prevention (CDC) in serial fourfold dilutions by indirect fluorescent antibody (IFA) testing for reactivity to *B. microti* (*11*), WA1 (*8*), and *B. divergens* antigens. The antigen sources were human isolates of *B. microti* and WA1 and a bovine isolate of *B. divergens* (the Purnell strain from the Republic of Ireland [*12*]) that had been passaged in gerbils (Mongolian jirds; *Meriones unguiculatus*) and adapted to culture in bovine erythrocytes. The serum specimens were also tested at the Clinical Institute of Hygiene of the University of Vienna by IFA for reactivity to *B. divergens* antigens (from a bovine isolate from Hanover, Germany, that had been passaged in jirds); the dilutions of serum that were tested were 1:16, 1:64, 1:256, 1:1,000, and 1:4,000.

### Animal Inoculation

Five jirds, which are competent hosts for *B. divergens* (*13*), were injected intraperitoneally with 0.5 mL of 1-day old, refrigerated, pretreatment blood from the Austrian patient. Animal experimentation guidelines were followed. The jirds were monitored periodically (at least weekly; 26 times in 17.5 weeks) for parasitemia by examination of Diff Quik-stained (DADE AG, Düdingen, Switzerland) smears of blood obtained either by tail snip or, at the end of the monitoring period, by cardiac puncture after anesthesia with ketamine. The blood obtained by cardiac puncture was also examined by polymerase chain reaction (PCR) (see below).

### DNA Extraction, Amplification, and Sequencing

DNA was extracted from EDTA-stabilized whole blood from the two patients by using the QIAamp DNA Blood Mini Kit (QIAGEN Inc., Valencia, CA); the DNA was stored at 4°C. The complete 18S ribosomal RNA (18S rRNA) gene was amplified by PCR, with a pair of generic apicomplexan 18S rRNA-specific primers: CRYPTOF, the forward primer (5′-AACCTGGTTGATCCTGCCAGT-3′), and CRYPTOR, the reverse primer (5′-GCTTGATCCTTCTGCAGGTTCACCTAC-3′). PCR was conducted with the AmpliTaq Gold DNA Polymerase (Applied Biosystems, Foster City, CA). The conditions for PCR included 95°C for 15 min, followed by 45 cycles of denaturation at 94°C for 30 s, annealing at 65°C for 30 s, and extension at 72°C for 1.5 min. Final extension was done at 72°C for 9 min, followed by a hold step at 4°C. Amplification products were purified by using the StrataPrep DNA Purification Kit (Stratagene, La Jolla, CA). In addition, DNA provided us that had been extracted from two isolates of *B. odocoilei* (i.e., the Brushy Creek and Engeling isolates [*14*]), a parasite of white-tailed deer (*Odocoileus virginianus*) (*15*,*16*), and from *B. divergens* [Purnell strain] [*12*]) was analyzed.

Both strands of the PCR products were sequenced by using a set of internal primers. Sequencing reactions were conducted with the ABI PRISM BigDye Terminator Cycle Sequencing Kit (Applied Biosystems), and reactions were analyzed on the ABI 377 or ABI 3100 automatic DNA sequencer (Applied Biosystems). The resulting sequences were assembled by using the program SeqMan II (DNASTAR, Inc., Madison, WI). The GenBank accession numbers for the complete sequences we generated of the 18S rRNA gene for the various organisms are as follows: *B. odocoilei*, AY046577; *B. divergens*, AY046576; and EU1, AY046575.

### Phylogenetic Analysis

**Figure 1:**
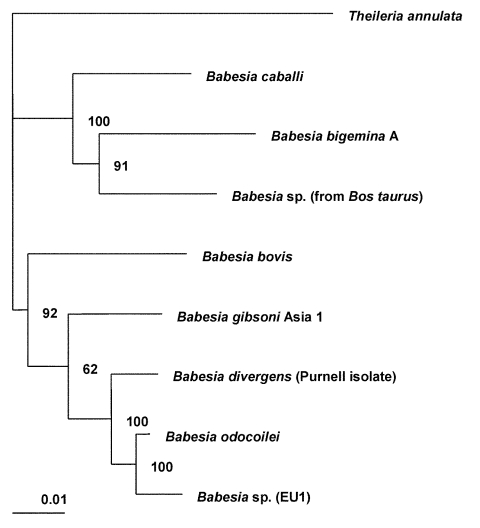
Phylogenetic tree for the complete 18S rRNA gene from selected *Babesia* spp. The tree was computed by using the quarter puzzling maximum likelihood method of the TREE-PUZZLE program and was oriented by using *Theileria annulata* as the outgroup. Numbers at the nodes indicate the quartet puzzling support for each internal branch. Scale bar indicates an evolutionary distance of 0.01 nucleotides per position in the sequence. Vertical distances are for clarity only. The GenBank accession numbers for the sequences used in the analysis are as follows: *Babesia bigemina* A, X59604; *B. bovis*, L19077; *B*. *caballi*, Z15104; *B*. *divergens* (Purnell isolate [*12*]), AY046576; *B. gibsoni* (genotype Asia 1), AF175300; *B*. *odocoilei* (Brushy Creek and Engeling isolates [*14*]), AY046577; *Babesia* sp. (isolated from *Bos taurus*), U09834; EU1 (the etiologic agent of infection in the two cases described here), AY046575; and *Theileria annulata*, M64243.

The complete sequences of the 18S rRNA genes for *B. bigemina*, *B. bovis*, *B. caballi*, *B. divergens*, *B. gibsoni*, *B. odocoilei*, and *Babesia* sp. (isolated from *Bos taurus*) were retrieved from the GenBank database (see Figure 1 legend for GenBank accession numbers) and aligned with the sequence for EU1 by using the program CLUSTAL W version 1.83 (*17*). The 18S rRNA sequence for *Theileria annulata* was included as the outgroup for the phylogenetic analysis. This analysis was performed with the following programs: the PHYLIP package, which includes versions 3.5 of CONSENSE, DNADIST, DNAML, NEIGHBOR, and SEQBOOT (*18*); and version 5.73c of TREE-PUZZLE (*19*). The phylogenetic trees inferred by these programs were drawn by using the program TreeView, version 1.6.6 (*20*). The trees were statistically evaluated by using bootstrap (*18*) and quartet puzzling methods (*19*).

### Case Reports

The Italian and Austrian patients were 55- and 56-year-old men, respectively, who had undergone splenectomy in the 1980s because of stage IA Hodgkin’s disease. The Italian patient had recently begun chemotherapy (Table footnote) for stage IIIA diffuse large B-cell lymphoma, which had been diagnosed in June 1998. Both men lived in small towns and hunted avocationally (Table); neither had pets. Only the Austrian patient recalled tick exposure—a tick bite while hunting about 2 weeks before he noticed his urine was dark. Neither patient had traveled extensively: the Italian patient had never left Italy, and the Austrian patient had been in Barbados (1998) and Turkey (1999).

**Table:** Characteristics of two men who had babesiosis in 1998 and 2000, respectively^a–c^

Characteristics	Italian patient	Austrian patient
**Residence and outdoor activities**	Lived in northern Italy, in a small town in the district of Romagna, on ~1 hectare of land; often hunted moles in his garden, even after he started chemotherapy	Lived in northeastern Austria, in a small town in the district of Krems Land, in the province of Lower Austria; had an off-site garden; often hunted in the Dunkelsteinerwald forest (usually wild boars, sometimes foxes and badgers)
**Clinical illness and general laboratory data**		
Initial clinical manifestations	Fever (39°C) and chills developed on October 14, 1998; hospitalized on October 18 because of fever, headache, confusion, jaundice, and dark urine (discharged on November 6)	Marked fatigue developed on July 23, 2000; dark urine, without dysuria, developed on July 24; hospitalized on July 25 (discharged on August 2)
Hematologic parameters^d^		
Hemoglobin (g/dL)	4.8	15^e^ (13.2 on July 27, 2000)
Leukocyte count (x10^9^/L)	4.4	7.3 (7.8, with 5% atypical lymphocytes, on July 26)
Platelet count (x10^9^/L)	71	15 (8 on July 27)
Values of serum chemistries^d^		
Lactate dehydrogenase (U/L)	7,877 (normal range 230–460)	994 (July 26, 2000) (normal range 120–240)
Total bilirubin (mg/dL)	3.2 (normal range 0.2–1.10)	3.27 (July 26) (normal range 0.2–1.0)
Indirect bilirubin (mg/dL)	2.4 (normal range 0.2–0.85)	2.36 (July 26) (normal range 0.0–1.0)
Direct (conjugated) bilirubin (mg/dL)	0.8 (normal range 0.0–0.25)	0.91 (July 26) (normal range 0.0–0.25)
Creatinine (mg/dL)	2.5 (normal range 0.50–1.20)	1.04 (normal range 0.5–1.3)
**Diagnosis of *Babesia* infection**		
Parasitemia level (% of erythrocytes that were infected) on first blood smear examined	~30% (October 24, 1998)	1.3% (July 25, 2000) (Figure 2)
Antibody titers in IFA testing for reactivity to *B. divergens* antigens^f^	Titers of 1:64 (specimen from October 28, 1998) and 1:256 (February 15, 1999) in testing at both CDC and the Clinical Institute of Hygiene of the University of Vienna	Titers of 1:256 (July 31, 2000) and 1:1,024 (August 8, 2000) in testing at CDC and titers of 1:64 (July 31) and 1:1,000 (August 8) in testing at the Clinical Institute of Hygiene of the University of Vienna
**Therapy for babesiosis**		
Antimicrobial therapy	Clindamycin (600 mg thrice daily, by intravenous infusion) and quinine sulfate (650 mg thrice daily, by mouth) for 15 days, from October 24, 2000 (i.e., 10 days after onset of fever), through November 7	Clindamycin (600 mg thrice daily), by intravenous infusion, for 8 days, from July 25, 2000 (i.e., 2 days after onset of symptoms), through August 1, and by mouth, for 15 days thereafter (through August 15)
Blood transfusions	11 U packed erythrocytes, from October 19–31, 1998^c,g^	None
Response to therapy	Fever resolved by day 3 of therapy; no parasites found by blood-smear examination after day 6 of therapy; negative PCR analysis of blood from February 15, 1999	Blood from August 8, 2000, negative by blood-smear examination but positive by PCR analysis; negative PCR analysis of blood from November 7, 2000, and February 8, 2001
Long-term follow-up	Non-Hodgkin’s lymphoma remitted during hospitalization in 1998, but the lymphoma relapsed in February 2000; no parasites were found on blood smears during subsequent chemotherapy	Remained well

^a^IFA, indirect fluorescent antibody; PCR, polymerase chain reaction; CDC, Centers for Disease Control and Prevention. ^b^Non-Hodgkin’s lymphoma developed in the Italian patient (diagnosis: June 1998). Chemotherapy, begun on September 23, 1998, was stopped prematurely on October 14, after he became febrile. His chemotherapeutic regimen included daily prednisone (75 mg) and weekly administration of various drugs in rotation. He received 4 of the intended 12 weeks of therapy, which included doxorubicin and cyclophosphamide during odd-numbered weeks (weeks 1 and 3) and vincristine and either methotrexate (week 2) or bleomycin (week 4) during even-numbered weeks. ^c^Although the possibility that he became infected by blood transfusion could not be excluded because he had been transfused before blood smears were examined, his febrile illness and hemolytic anemia preceded the transfusions. ^d^Laboratory values were from hospital admission (October 18, 1998, for the Italian patient, and July 25, 2000, for the Austrian patient), unless otherwise specified. Values for the Austrian patient are from testing performed at the hospital to which he was transferred after a brief (<24-hour) stay at a local hospital. ^e^Earlier on July 25, at a local hospital, his hemoglobin value was 16.2 g/dL, which had been his approximate baseline value during the previous 10 months. ^f^IFA testing of serum specimens from both patients was negative for antibodies to *B. microti*. A specimen from the Italian patient (February 15, 1999) was negative for antibodies to WA1. ^g^Plasma exchange was performed on October 23, when he mistakenly was thought to have thrombotic th

The two cases ranged in severity from quite mild (Austrian case) to moderately severe (Italian case). The salient clinical details of their cases and the relevant laboratory values are provided in the Table. Fever occurred only in the Italian patient (maximum of 39°C), which initially was considered a reaction to one of his chemotherapeutic agents (i.e., bleomycin). He also had marked anemia, for which he received blood transfusions (Table). Both patients had thrombocytopenia, elevated serum lactate dehydrogenase and bilirubin values, and dark urine from hemoglobinuria. The Italian patient’s creatinine value also was elevated.

**Figure 2:**
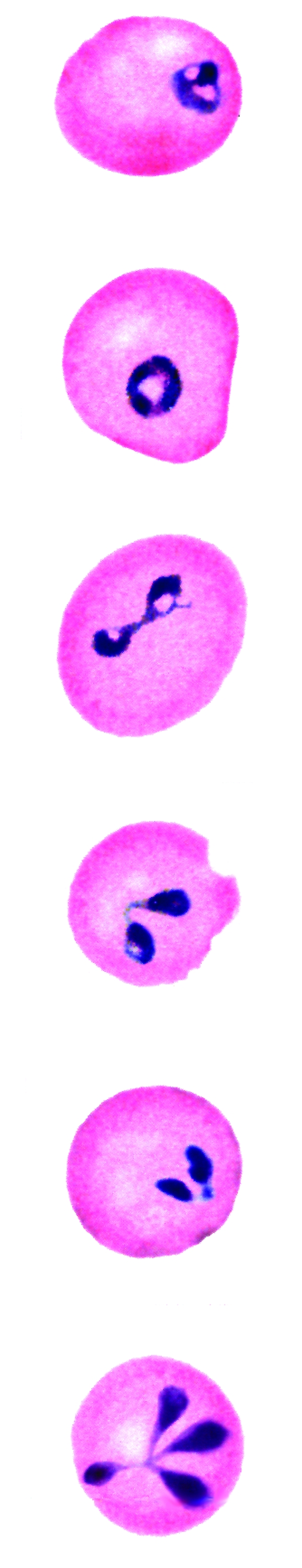
Panel of computer-generated electronic images of photomicrographs of *Babesia*-infected erythrocytes on a Giemsa-stained smear of peripheral blood from the patient who became infected in Austria. The electronic images were edited for uniformity of color, without changing the form or size of the organisms. The image on the far right shows a tetrad (Maltese-cross form). Three glass slides of the actual blood films have been deposited in the Oberösterreichisches Landesmuseum, Biologiezentrum, Linz (i.e., Biology Center of the Upper Austrian Museum, Linz), with the accession number 2002/9. The slides are labeled “*Babesia* sp. (EU1), patient 001, Austria, Krems Land, July 25, 2000.”

In both cases, babesiosis was diagnosed by noting parasitic inclusions in erythrocytes on peripheral blood smears (Table; Figure 2). The intervals between onset of the symptoms that ultimately were attributed to babesiosis and confirmation of the diagnosis ranged from 2 days (Austrian case) to 10 days (Italian case). Subsequent testing of serum specimens from both patients showed IFA reactivity to *B. divergens* but not to *B. microti* antigens; serum from the Italian patient was also tested for reactivity to WA1 antigens and was negative. Attempts to obtain an isolate of the parasite that infected the Austrian patient, by injecting specimens of his blood into jirds, were unsuccessful; the smears of blood from periodic tail snips and PCR analysis of blood obtained by cardiac puncture of the jirds were negative. Both patients responded to antimicrobial therapy for babesiosis: the Austrian patient was treated with clindamycin, and the Italian patient was treated with both clindamycin and quinine (Table).

### Molecular Data

Amplification of the complete 18S rRNA gene, by using generic protozoan primers and the *Babesia* DNA extracted from the patients as the templates, yielded a specific product of approximately 1,700 base pairs for each patient. Sequence analysis showed that the 18S rRNA gene was 1,727 bases long and that the PCR products from the two patients had identical sequences. BLAST (available from: URL: http://www.ncbi.nlm.nih.gov/BLAST/) search showed that the sequence, although clearly from a *Babesia* sp., was not identical to any complete 18S rRNA sequences in the GenBank database. In phylogenetic analysis, EU1 clusters together with *B. odocoilei*, and these two organisms form a sister group with *B. divergens* (Figure 1). The clustering of these organisms was identical, regardless of which phylogenetic method was used. The associations were strongly supported statistically. Support for the internal branch leading to the *B. divergens*, *B. odocoilei*, and EU1 group was 100% with both quartet puzzling and bootstrap distance analysis; for the internal branch separating *B. divergens* from *B. odocoilei* and EU1, the support was 100% for quartet puzzling and 88% for bootstrapped distance analysis. The alignment of the sequences used to construct the phylogenetic tree (Figure 1) is available from the authors upon request.

Because the complete 18S rRNA sequences in GenBank that were previously determined for various bovine isolates of *B. divergens* were not identical, we reanalyzed the complete 18S rRNA gene from isolates (cultures or DNA) from Ireland (Purnell [*12*]; GenBank accession no. U16370), Germany (U07885 [*21*]), and Northern Ireland (Z48751) that were provided to us. The sequences of the 18S rRNA gene we obtained for these isolates were identical, which suggests that no variability is present in this gene among geographically distinct bovine isolates of *B. divergens* (Slemenda et al., unpub. data). In contrast, the EU1 and *B. divergens* 18S rRNA sequences differed by 31 bases.

Similarly, our sequences of the 18S rRNA gene for both isolates of *B. odocoilei* (i.e., Brushy Creek and Engeling isolates) were identical to each other (GenBank accession no. AY046577) and to the *B. odocoilei* sequence with the GenBank accession no. U16369 (*14*). The EU1 and *B. odocoilei* 18S rRNA sequences differed by 29 bases.

## Discussion

We investigated the first reported human cases of babesiosis in Italy and Austria and have provided molecular evidence that the etiologic agent was a previously uncharacterized *Babesia* organism, which we refer to here as EU1. The organism was found in countries in Europe not previously known to have zoonotic babesiosis and had novel molecular characteristics for the genetic marker we analyzed, the complete 18S rRNA gene. Sequence analysis of this gene provides an objective and precise means of species identification and phylogenetic classification. The DNA sequences of the 18S rRNA gene were identical for the *Babesia* organisms from the two patients, which indicates that they were infected with the same organism. Each of the organisms was sequenced in a different country, which indicates that the findings were not artifactual.

The phylogenetic analysis (Figure 1) indicates that EU1 is most closely related to but distinct from *B. odocoilei*, which infects white-tailed deer (*15*,*16*) and is not known to infect humans. EU1 and *B*. *odocoilei* form a sister group to *B. divergens*, a bovine parasite that has been considered the main *Babesia* pathogen of humans in Europe. We have demonstrated that no variability exists in the 18S rRNA sequences among several geographically distinct bovine isolates of *B. divergens* (Slemenda et al., unpub. data), which is the organism to which the name *B. divergens* legitimately applies, and showed that EU1 clearly is not *B. divergens*.

EU1 is also distinct from the MO1 parasite, which caused a fatal human case of babesiosis in Missouri in 1992 and was thought then by the investigators to be *B. divergens*–like but distinct from it (*10*). The sequence provided in the publication about MO1 (*10*) was for only a 128–base pair fragment; in that region, the EU1 and MO1 sequences differ by four bases, and three positions in the MO1 sequence were unresolved.

The DNA sequences available in GenBank for *B. divergens* in Europe are from cattle not humans. To our knowledge, molecular data have been reported for only one of the purported human cases of *B. divergens* infection in Europe, a case on the Canary Islands (*22*,*23*). However, the data were for an incomplete 18S rRNA sequence (GenBank accession no. AF435415), and therefore were not suitable for the phylogenetic analysis we performed of complete 18S rRNA sequences. Nevertheless, the sequence for the case on the Canary Islands differs by 18 bases with the sequence for EU1 and by 1 base with the *B. divergens* sequence from cattle (AY046576) in the 369-base-long region of the gene that could be compared.

In the absence of molecular data, we are not certain which organisms have caused the human cases of babesiosis in Europe that have been attributed to *B. divergens*. The evidence that particular human cases were caused by *B. divergens* has varied in quantity, quality, and type. The evidence typically has included various combinations of morphologic data, from examination of blood smears; serologic data (usually, but not always, from IFA testing); and data concerning whether jirds or cattle injected with the patient’s blood become parasitemic. Although these techniques are useful for detecting *Babesia* infection, they do not necessarily provide reliable species identification (e.g., because of serologic cross-reactivity between EU1 and *B. divergens* in IFA testing [Table]). Although some of the human cases attributed to *B. divergens* may truly have been caused by the bovine *B. divergens*, others might have been caused by EU1. The cases of EU1 infection we reported likely would have been attributed to *B. divergens* had only the traditional methods of characterization, without molecular analysis, been used.

Our molecular characterization also showed that EU1 is not closely related to the other *Babesia* (or *Babesia*-like) agents known to have infected humans (most notably, *B. microti* and the WA1- and CA1-type parasites). *B. microti*, together with *B. rodhaini*, *Cytauxzoon felis*, and *B. equi*, is ancestral to the *Theileria* spp. and perhaps also to the *Babesia* sensu stricto group (depending on which tree topology is used) (*24*). Reclassification of the *B. microti* group to a new family has been proposed (*24*). The WA1- and CA1-type parasites, which have caused human cases of babesiosis in the western United States (*8*,*9*), also form a well-defined group, whose position in the phylogeny of the piroplasms is uncertain (*25*).

Although EU1 represents a zoonotic pathogen with previously unreported molecular characteristics, whether it represents a new species per se awaits further evidence.^1^ EU1 might constitute a new species in the sense that it was never previously recognized or characterized in any way or one that was characterized but not with molecular data (e.g., was misnamed *B. divergens* or some other *Babesia* sp.). Because DNA sequence data are not available for most of the *Babesia* spp. found over the past century in nonhuman animals and because data about the morphologic features and host specificity of a parasite are inadequate for definitive species identification, we cannot exclude the possibility that EU1 is one of the many previously described *Babesia* spp. of nonhuman animals, some named and some not, that were not known to be zoonotic.

Although the serologic cross-reactivity between EU1 and *B. divergens* could have resulted in diagnostic confusion in the past, cross-reactivity between these two organisms also could be advantageous. The *B. divergens* IFA could be a useful tool for testing serum from persons who might be infected with EU1 or who participate in serosurveys to determine the prevalence and geographic distribution of EU1 infection. Unfortunately, our attempts to obtain an isolate of EU1 by inoculation of jirds were unsuccessful. One consequence is that we did not generate the homologous antigen needed for development of an IFA assay for EU1. Therefore, we could not contrast the degree of reactivity of our patients’ serum specimens with antigens from EU1 and *B. divergens*.

The importance of determining whether the etiologic agent of a particular case of babesiosis is EU1 rather than *B. divergens* or some other *Babesia* sp. depends in part on whether the clinical manifestations of infection and the response to antimicrobial therapy differ. We cannot generalize about such issues from two cases of infection with EU1*.* However, the range in severity of the two cases, from quite mild (Austrian case) to moderately severe (Italian case), is of interest, particularly because the two patients were similar in some respects (i.e., both were asplenic men in their mid-fifties). Factors that likely placed the Italian patient at increased risk for a more severe case included immunosuppressive chemotherapy for lymphoma and the 10-day interval between the onset of fever and the diagnosis of babesiosis (Table).

Largely from data for *B. microti* infection in the United States, combination therapy with either clindamycin and quinine or atovaquone and azithromycin is recommended for treatment of babesiosis (*28*), with the addition of exchange transfusion in some situations in severely ill patients. The Austrian patient, whose case was mild, was treated with clindamycin only. Some in vitro data and anecdotal clinical data for purported zoonotic cases of *B. divergens* infection suggest that therapy with clindamycin alone, in combination with exchange transfusion, when indicated, might be effective for treating *B. divergens* infection (*4*,*29*,*30*). However, no clinical trials in humans have evaluated the effectiveness of any antimicrobial regimens for treatment of *Babesia* infection not caused by *B. microti*.

The public health importance of infection with EU1, including such factors as its biology, geographic distribution, ecology, prevalence, risk factors for infection and disease, clinical manifestations, tick vector, and animal reservoir host(s), is not yet known and may take years to determine. The Italian patient likely became infected in a garden habitat and the Austrian patient in a garden or forest habitat. Of interest, an incomplete 18S rRNA sequence (GenBank accession no. AF373333) for a *Babesia* sp. found in *I. ricinus* ticks was recently reported by investigators in Slovenia (*31*), which borders Italy and Austria. The sequence was reported for only 364 bases and corresponds to positions 433–796 of the complete 18S rRNA sequence for EU1. In this part of the gene, the sequences for the organisms from the Slovenian ticks and EU1 are identical. However, the relatedness of the two organisms cannot be determined without the complete 18S rRNA sequence for the organism from the ticks. The occurrence of two identified cases of EU1 infection in humans in different countries (i.e., Italy and Austria) and years (i.e., 1998 and 2000) indicates that EU1 is not restricted to one geographic area or time. Increased vigilance for zoonotic infection with novel vector-borne pathogens is needed.

## Footnotes

1 The issue of what constitutes a new or newly described species requires periodic reevaluation as the techniques for characterizing microbes improve. Although the advent of molecular biology/phylogeny has made it possible to characterize organisms more precisely, the issue of how large a genetic difference in a particular gene(s) constitutes a new or different species is controversial. Bacterial taxonomy is a much more active and advanced field (*26*,*27*) than that for characterizing protozoa, in part because of the increasingly large numbers of bacteria being proposed as new species and the challenges posed by such possibilities as genetic rearrangements. In the recommendations published in 2002 by an ad hoc committee that reevaluated the species definition for bacteria (*26*), scientists were encouraged to use the “*Candidatus”* concept (i.e., to propose candidates for newly described bacterial species) for organisms that had been well-characterized, including the sequencing of the small subunit RNA gene, but had not yet been cultured. As noted above, we have referred to the protozoan we characterized as EU1. We have not claimed it as a newly identified species, despite having complete, identical, and novel 18S rRNA sequences for the organism from two patients, who were separated in time and space, and having done the sequence analysis for the two cases in different countries. However, although the *Candidatus* concept per se does not formally exist now for protozoa, on the basis of the precedent from the field of bacterial taxonomy, we propose that EU1 be considered a candidate species. If additional evidence supports the conclusion that the organism indeed constitutes a newly described species of the *Babesia* genus, we would favor the name *Babesia venatorum,* which now does not constitute an official name. We chose this name because the patients whose cases we reported were avocational hunters; “venator” is the Latin word for “hunter” (“venatorum,” the plural genitive case, means “of the hunters”).
